# The Use of Cancer-Specific Patient-Centered Technologies Among Underserved Populations in the United States: Systematic Review

**DOI:** 10.2196/10256

**Published:** 2019-04-23

**Authors:** Will L Tarver, David A Haggstrom

**Affiliations:** 1 VA Health Services Research and Development Center for Health Information & Communication Richard L Roudebush VA Medical Center Indianapolis, IN United States; 2 Department of Health Policy & Management Fairbanks School of Public Health Indiana University Indianapolis, IN United States; 3 Division of General Internal Medicine and Geriatrics School of Medicine Indiana University Indianapolis, IN United States; 4 Center for Health Services Research Regenstrief Institute Indianapolis, IN United States

**Keywords:** underserved populations, medical informatics, cancer

## Abstract

**Background:**

In the United States, more than 1.6 million new cases of cancer are estimated to be diagnosed each year. However, the burden of cancer among the US population is not shared equally, with racial and ethnic minorities and lower-income populations having a higher cancer burden compared with their counterparts. For example, African Americans have the highest mortality rates and shortest survival rates for most cancers compared with other racial or ethnic groups in the United States. A wide range of technologies (eg, internet-based [electronic health, eHealth] technologies, mobile [mobile health, mHealth] apps, and telemedicine) available to patients are designed to improve their access to care and empower them to participate actively in their care, providing a means to reduce health care disparities; however, little is known of their use among underserved populations.

**Objective:**

The aim of this study was to systematically review the current evidence on the use of cancer-specific patient-centered technologies among various underserved populations.

**Methods:**

Computer-based search was conducted in the following academic databases: (1) PubMed (cancer subset), (2) MEDLINE, (3) PsycINFO, and (4) CINAHL. We included studies that were peer-reviewed, published in the English language, and conducted in the United States. Each study was individually assessed for relevance, with any disagreements being reconciled by consensus. We used a 3-step inclusion process in which we examined study titles, abstracts, and full-text papers for assessment of inclusion criteria. We systematically extracted information from each paper meeting our inclusion criteria.

**Results:**

This review includes 71 papers that use patient-centered technologies that primarily targeted African Americans (n=31), rural populations (n=14), and Hispanics (n=12). A majority of studies used eHealth technologies (n=41) finding them to be leading sources of cancer-related health information and significantly improving outcomes such as screening among nonadherent individuals and increasing knowledge about cancer and cancer screening. Studies on mHealth found that participants reported overall favorable responses to receiving health information via short message service (SMS) text message; however, challenges were experienced with respect to lack of knowledge of how to text among some participants. More complex mobile technologies (eg, a tablet-based risk assessment tool) were also found favorable to use and acceptable among underserved populations; however, they also resulted in more significant barriers, for example, participants expressed concerns regarding security and unfamiliarity with the technology and preferred further instruction and assistance in its use.

**Conclusions:**

There is a growing body of literature exploring patient-centered technology and its influence on care of underserved populations. In this review, we find that these technologies seem to be effective, especially when tailored, in improving patient and care-related outcomes. Despite the potential of patient-centered technologies and the receptivity of underserved populations, challenges still exist with respect to their effective use and usability.

## Introduction

### Background

In the United States, more than 1.6 million new cases of cancer are estimated to be diagnosed each year [1]; however, the burden of cancer among the US population is not shared equally. Medically underserved populations are defined as groups with economic, cultural, or linguistic barriers to medical care services [2]. These groups include racial and ethnic minorities and individuals of lower socioeconomic status [3] who have a higher cancer burden compared with their counterparts, which can be partially attributed to differences in the access to, and quality of, care they receive [4-6]. A wide range of technologies is available to patients, which have the potential to improve access to care and empower individuals to participate more actively in their care [7,8]. These technologies include personal health records (PHRs) [9], internet-based (eHealth) technologies [10,11], mobile (mHealth) apps [12], and telemedicine [13]. For example, there is evidence that patient-centered technologies (also commonly referred to as *consumer health information technologies*) provide patient-centered care by increasing patients’ quality of health care [14], improving communication with providers [13,15-17], providing tailored education and lifestyle messages [14,18], and promoting self-management of health care [19]. Among other cancer health promotion activities, these technologies educate individuals on the benefits of cancer screening, enable individuals to receive reminders for cancer screening and follow-up, and provide tailored decision aids for cancer care. These health information technologies have also been proposed as a means to reduce health care disparities [8,20-22]. The Institute of Medicine identified the internet and computers as critical vehicles to deliver health information to reach diverse populations of cancer patients and survivors [23,24], including low-literacy and low-income African Americans [25-28]. Research is crucial to understand the use and impact of these technologies among underserved populations for the purposes of cancer health promotion, but the medical literature has not been systematically reviewed to understand these patterns or outcomes.

To date, reviews on the use of patient-centered technologies have largely focused on the general population. For example, a recent review by Kim and Nahm [29] found several benefits to the use of patient-accessible PHRs, including consumer empowerment, improved patient-provider communication, increased access to data during times of emergency, improved chronic disease management, and increased likelihood of behavior change. Several concerns were also raised regarding the broader dissemination of PHRs, including data privacy and security, data accuracy, health literacy, and the digital divide. With regard to mobile technologies, Krishna et al [30] conducted a review and found significant improvements in medication adherence, smoking quit rates, self-efficacy, and other health outcomes (eg, asthma symptoms, blood sugar control, and stress levels). Limited attention has also been given to the potential of patient-accessible PHRs among specific disease classes [31,32]; however, Price et al [33] found PHR interventions targeting asthma, diabetes, fertility, glaucoma, HIV, hyperlipidemia, and hypertension (but not cancer) to have beneficial effects such as better quality of care, improved access to care, and increased productivity. In another review, Bennet et al [34] found that racial or ethnic minority populations have been targeted with interventions to facilitate weight loss. Overall, internet-based technologies (eHealth) were only able to affect short-term weight loss, whereas mobile technologies (mHealth) provided no benefit [34]. Montague et al [35] also found that technologies can positively affect the health of the underserved if they are effectively tailored, but little is known about how to effectively tailor cancer-specific technologies to this population. Although eHealth and mHealth studies have also explored the use and adoption of these technologies [36-40] and have been shown to increase screening for cancer [41] and knowledge of cancer and cancer screening [42], these studies have not been systematically reviewed and synthesized to date.

The purpose of this study is to systematically review current evidence on the use of cancer-specific patient-centered technologies among underserved populations. This review contributes to both the informatics and cancer health disparities literature by seeking to address the following issues: (1) to understand the effect or impact of patient-centered technologies on the health or health care outcomes of underserved populations, (2) to understand the use, usability, and acceptance of patient-centered technologies and efforts to tailor their design to improve cancer care among underserved populations, (3) to understand the barriers and facilitators to patient-centered technology use for different populations, and (4) to propose directions for future research based on the current literature.

### Conceptual Framework

Patient-centered technology use by underserved populations is influenced by multiple factors. For purposes of this review, we have adapted an existing health services research framework to organize the factors that influence the use and acceptance of information technology among individuals. Originally developed with the organization in mind, the unified theory of acceptance and use of technology (UTAUT) [43] sought to understand the critical factors related to the prediction of behavioral intention to use technologies within the organizational context.

**Figure 1:**
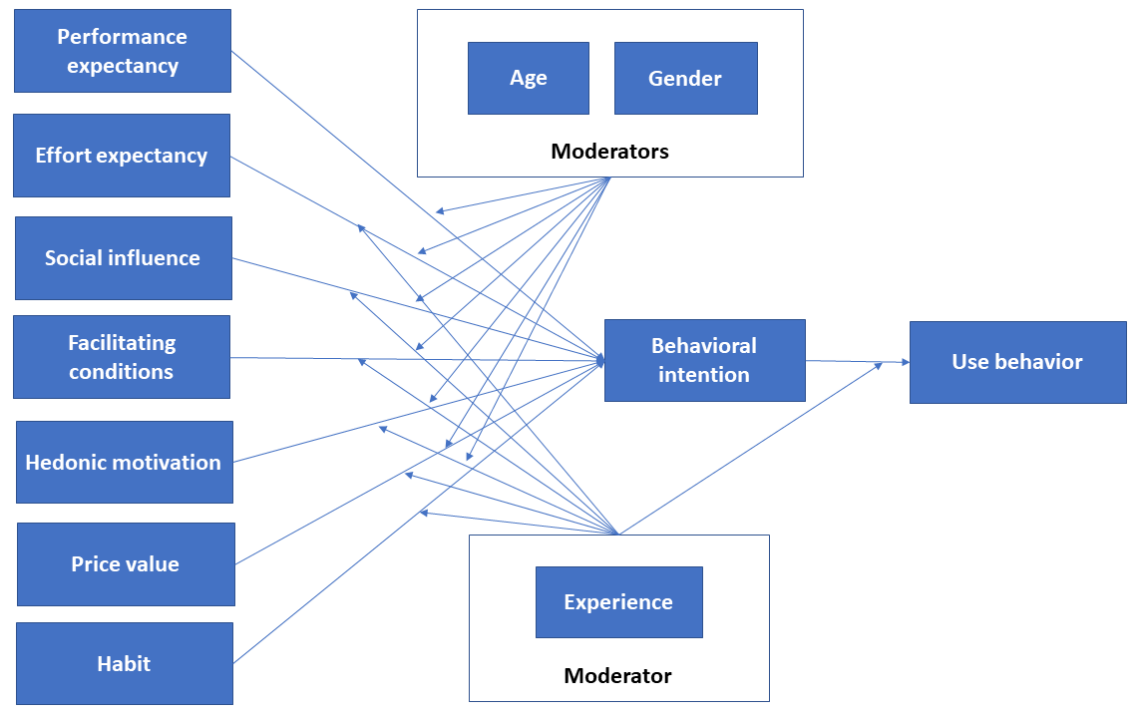
Venkatesh et al's [44] consumer acceptance model of the unified theory of acceptance and use of technology.

According to the UTAUT, there are 3 constructs (ie, performance expectancy, effort expectancy, and social influence) that are considered direct determinants of the intention to use technology (see Table 1 and Figure 1). *Performance expectancy* refers to the degree to which using a technology will provide benefits to consumers in performing certain activities. In the health context, benefit examples may include managing chronic conditions or receiving health information to facilitate behavior change. *Effort expectancy* refers to the degree of ease associated with the consumers’ use of technology (ie, usability). *Social influence* refers to the extent to which consumers perceive important others, such as their family and friends, to believe they should use a particular technology. Separately, there are 2 constructs within this framework (ie, intention and facilitating conditions) that are considered direct determinants of technology usage behavior, with facilitating conditions referring to the perception of resources and support available to perform a behavior. In addition, there were 4 moderators embedded in this original framework, which contributed to understanding the acceptance of technology by individuals (ie, age, gender, experience, and voluntariness of use).

To tailor this theory to the consumer use of technology, Venkatesh et al developed UTAUT2 [44]. Given the focus of this study, it seems especially apt to use this tailored theory to understand minorities’ use of patient-centered technologies. Under this remodeled framework, 4 key constructs on the general and consumer adoption and use of technologies have been identified and incorporated into UTAUT. These additional constructs are (1) hedonic motivation (the fun or pleasure derived from using a technology), (2) price value (the monetary cost of use on the individual), (3) experience (the passage of time from initial use of the technology), and (4) habit (the extent to which an individual believes the behavior to be automatic).

**Table 1:** Constructs of the consumer acceptance model of the unified theory of acceptance and use of technology (UTAUT).

Constructs		Operational definitions
**UTAUT constructs**		
	Performance expectancy	The degree to which using a technology will provide benefits to consumers in performing certain activities
	Effort expectancy	The degree of ease associated with the consumers’ use of technology
	Social influence	The extent to which consumers perceive the important others (family and friends) believe they should use a particular technology
**UTAUT2 constructs**		
	Hedonic motivation	The fun or pleasure derived from using a technology
	Price value	The monetary cost of use on the individual
	Experience	The passage of time from initial use of the technology
	Habit	The extent to which an individual believes the behavior to be automatic

## Methods

### Search Strategy

Recommendations of the statements on enhancing transparency in reporting the synthesis of qualitative research [45] and the preferred reporting items for systematic reviews and meta-analyses [46] were followed. Web-based search was conducted in the following academic databases: (1) PubMed (cancer subset), (2) MEDLINE, (3) PsycINFO, and (4) CINAHL. To optimize search results, we used various combinations of keywords taken from the existing literature and Medical Subject Headings terms. A complete list of search terms is provided in Table 2. Finally, we identified additional studies using a snowball searching technique whereby the reference lists of studies that met our inclusion criteria were examined.

### Inclusion Criteria

We identified papers that appeared in peer-reviewed journals and were published in the English language up to October of 2016. We included both qualitative and quantitative studies and excluded nonempirical studies such as commentaries as well as international studies. Similar to other reviews [35], we limited eligible studies to those conducted in the United States because sociocultural differences in the United States may be unique from other countries. Studies were included if they assessed patient-centered technologies among underserved populations. The specific topics of interest for this review included (1) the effect of these technologies on the health or health care outcome studied; (2) the use, usability, and acceptance of these technologies and efforts to tailor their design to populations of interest; (3) facilitators and barriers to the use of patient-centered technologies among underserved populations; and (4) implementation lessons learned from studies assessing these technologies among underserved populations. Studies were included if they focused exclusively on underserved populations or underserved groups represented at least 40% of their sample size. To categorize the health information technology (HIT) apps of interest in this study, the following definitions were used.

### Definitions

#### Electronic Health

Although no standard definition for eHealth exists [11], the term eHealth has been used broadly in the literature to refer to technologies ranging from CD-ROMs to the internet. For purposes of this study, eHealth is defined as “...the use of emerging information and communication technology, especially the internet, to improve or enable health and health care” [47].

#### Mobile Health

mHealth technologies are defined as “...a personalized and interactive service whose main goal is to provide ubiquitous and universal access to medical advice and information to any users at any time over a mobile platform” [48]. mHealth technologies can include the use of cell phones, smartphones, and tablets by patients or health care providers.

#### Telemedicine

Telemedicine has been defined as “...a branch of e-health that uses communications networks for delivery of health care services and medical education from one geographical location to another” [49]. The concept of distance is essential, for example, telemedicine can improve access to care to rural populations by eliminating distance as a barrier.

### Study Selection

**Figure 2:**
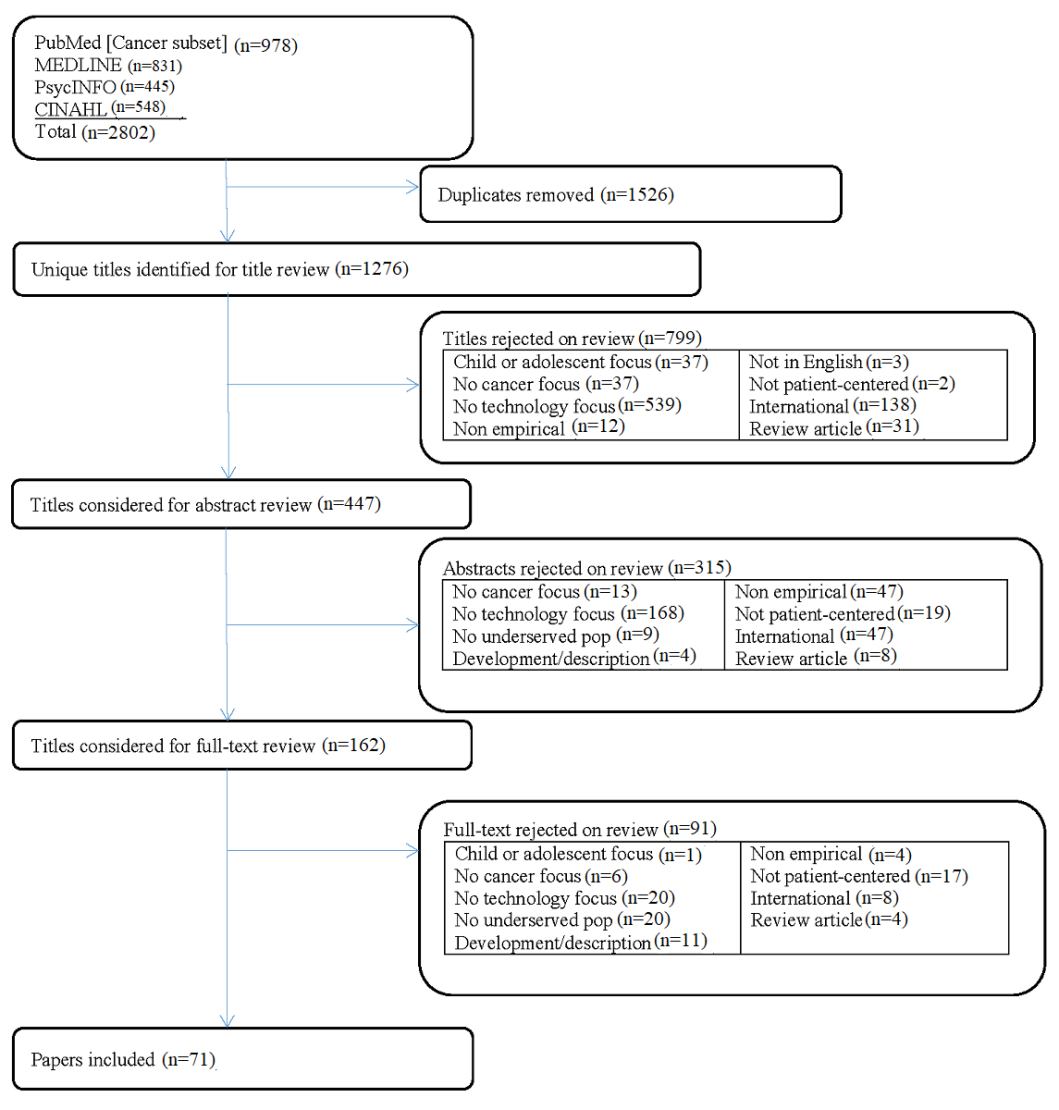
Systematic review flowchart. pop: population.

Each study was individually assessed for relevance. Any disagreements between reviewers were reconciled by consensus. We used a 3-step inclusion process, which is illustrated in Figure 2. In step 1, we examined paper titles and excluded papers that clearly did not have a focus on either patient-centered technologies or cancer care. In step 2, the remaining citation abstracts were retrieved. Similarly, we then excluded paper abstracts that clearly did not have a focus on either patient-centered technologies or cancer care. Finally, the full-text papers of the remaining citations were obtained for independent assessment of all inclusion criteria.

**Table 2:** Operationalization of the search terms.

Category	Search terms
Cancer^a^	Cancer, neoplasms
Underserved populations	Ethnic^b^, race, racial, disparity^b^, minority^b^, underserved, rural, hispanic^b^, mexican^b^, latino^b^, african^b^, black^b^, Asian, american indian^b^, alaskan native^b^, native american^b^, inuit^b^ or pacific islander^b^
Health information technology	health information technology, health it, electronic health records, electronic health record^b^, electronic medical record^b^, personal health record^b^, personal medical record^b^, patient accessible record^b^, patient portal^b^, patient internet portal^b^, decision support^b^, clinical reminder^b^, electronic reminder^b^, reminder system^b^, m-health, mhealth, mobile technolog^b^, mobile health, cell phone^b^, cellular phone^b^, smartphone^b^, mobile phone^b^, mobile device^b^, text message^b^, cd-rom, dvd, computer based, computer-based, internet-based, web-based, web based, e-health, ehealth, tablet, tailored, telemedicine, telehealth, teleoncology

^a^Search terms within each category are combined with OR. Search terms between categories are combined with AND. Some terms were truncated.

^b^Truncation of search term to capture keywords with the same stem.

### Data Extraction

Information systematically extracted from the papers included the following: study design, including the targeted cancer and/or stage of the cancer care continuum [50]. The continuum of cancer care refers to prevention, detection, diagnosis, treatment, survivorship, and end-of-life care [51]. In addition, we extracted information on the underserved population of interest; whether the patient-centered technology focused on healthy individuals, cancer patients or survivors, caregivers, or health care providers; sample size; the type of patient-centered technology used; the study outcome of interest; and whether there was any evidence of tailoring when it came to the technology intervention. Tailoring is defined as “Any combination of information or change strategies intended to reach one specific person, based on characteristics that are unique to that person, related to the outcome of interest, and have been derived from an individual assessment” [52]. Moreover, when available we described the technology’s use (whether patients adopt the tool), usability (the patient’s experience using the tool), and usefulness (the extent to which it meets the patient’s needs). Finally, we described barriers and facilitators to the use of the technologies reported in the paper.

## Results

### Studies Included

Our keyword search identified an initial yield of 1276 nonduplicative studies (Figure 2). The primary reasons for exclusion are also identified in Figure 2. After applying the exclusion criteria in review of the titles and abstracts, 71 studies were included in the systematic review (marked with an asterisk in the reference list), published between 1995 and 2016.

### Study Characteristics

The characteristics of the studies are summarized in Table 3. Studies varied with respect to the underserved population targeted, the technology used, and the cancer type of interest. A large proportion of studies included in our review target blacks or African Americans (31/71, 44%) followed by rural populations (14/71, 20%). More than half of the included studies assessed eHealth technologies (41/71, 58%). Moreover, the largest proportion of studies focused on breast cancer (26/71, 37%). In addition, the largest proportion of technological outcomes assessed was use of technology (20/71, 2%), whereas knowledge (15/71, 21%) was the largest proportion of health outcomes assessed. Observational studies represented the largest proportion of studies included in our review (32/71, 45%). Overall, 15 studies followed an experimental design (15/71, 21%), whereas the remaining 24 studies were either qualitative (13/71, 18%) or mixed-methods (11/71, 16%).

To provide a consistent structure, the remainder of the Results section is organized as follows. Study summaries are stratified by the type of patient-centered technology. Within each patient-centered technology section, we then further stratify by study design: experimental (Multimedia Appendix 1), observational (Multimedia Appendix 2), and qualitative studies (Multimedia Appendix 3).

### Theme 1. The Effect of Use on Clinical and Other Outcomes

#### Electronic Health

In total, 10 eHealth studies were identified using an experimental design, with 9 of these using a randomized controlled trial (RCT) [41,53-61] (Multimedia Appendix 1). These studies primarily targeted African Americans (n=7) as well as colorectal (n=6) and breast (n=4) cancer. Findings showed positive impacts of eHealth interventions, ranging from computer-assisted programs and Web-based decision aids to tailored, interactive soap operas. The most common health outcomes assessed included knowledge (n=5) and screening uptake (n=3), with each study showing statistically significant results in the outcomes.

For example, Champion et al [41] used an RCT to compare the efficacy of 3 interventions in promoting routine mammography screening among low-income African American women. This study found that an interactive computer-assisted instruction program produced the greatest adherence to mammography (40.0%) compared with participants receiving an educational pamphlet (32.1%) or a culturally appropriate video (24.6%). Jibaja et al [57] used an interactive soap-opera format to promote the early detection of breast cancer among high-risk Hispanic women. The use of this culturally tailored, computer-based educational program was found to significantly increase breast cancer screening knowledge and beliefs relative to a comparison group.

#### Mobile Health

We identified 3 mHealth studies using an experimental design [62-64], with 2 studies (66.7%) showing statistically significant results in the health outcome. Using a quasi-experimental design, Lee et al [64] tested a tailored interactive 7-day short message service (SMS) text message intervention designed to increase knowledge and vaccination of human papillomavirus (HPV). This study found a significant increase in knowledge and intent to get vaccinated. In addition, HPV vaccination uptake increased by 30% among participants in the intervention. Targeting a Hispanic population for CRC screening, Fernandez et al [63] used an RCT to compare (1) a tailored interactive multimedia intervention, (2) a lay health worker delivered media print intervention, and (3) a no intervention control group. No statistically significant differences were found among the study arms. Among a population with advanced prostate cancer that included a significant proportion of African Americans (40.5%), Yanez et al [62] found that a Web-based psychosocial intervention delivered via a tablet achieved good retention (>85%) and attendance rates (>70%) and received favorable evaluations (mean score: 4/5) and exit surveys (mean score: 3.6/4). The intervention also reduced depressive symptoms (43.37 vs 47.29, *P*=.03) and improved relaxation self-efficacy (2.43 vs 1.11, *P*<.01) for men who completed the study.

We also identified 2 additional studies using a mixed-methods approach with an experimental component. Among a sample of Korean American women, a 7-day mobile phone text message–based cervical cancer screening intervention significantly increased participants’ knowledge of cervical cancer and screening recommendations as well as the uptake of cervical cancer screening [42]. In a sample of Spanish-speaking Latina women seeking care at a federally qualified health center, text messaging reduced the number of days between an abnormal mammogram and participants’ return for follow-up compared with women who did not receive text message notifications [65].

#### Telemedicine

We identified 2 telemedicine studies using an experimental design, with each study showing statistically significant results in the health outcomes [66,67]. Kroenke et al [67], using an RCT design, found that telecare management improved depression and pain outcomes in both urban and rural cancer patients. In another RCT, telegenetics was compared with in-person cancer genetic counseling in terms of its impact on attendance, patient satisfaction, and cost [66]. This study found that although costs were significantly less, telegenetics did not differ in patient satisfaction from in-person genetic counseling; however, patients seeking in-person genetic counseling were more likely to attend counseling sessions.

**Table 3:** Characteristics of studies included in this review (N=71).

Characteristics		Total, n (%)
**Underserved population**		
	American Indian or Alaskan native	3 (4)
	Asian	6 (8)
	Black or African American	31 (44)
	Hispanic	12 (17)
	Diverse pop	4 (6)
	Low income	6 (9)
	Rural	14 (20)
**Patient-centered technology**		
	Computer- or internet-based technology (eHealth)	41 (58)
	Mobile app (mHealth)	15 (21)
	eHealth and mHealth	5 (7)
	Telemedicine	10 (14)
**Cancer type**		
	Breast	26 (37)
	Cervical	4 (6)
	Colorectal	12 (17)
	Lung	1 (1)
	Ovarian	1 (1)
	Prostate	9 (13)
	Cancer (not specific)	18 (25)
**Technology outcomes**		
	Use	20 (28)
	Usefulness	5 (7)
	Usability or acceptability	18 (25)
	Design or implementation	6 (9)
	Satisfaction	5 (7)
**Health outcomes**		
	Communication	1 (1)
	Decision making	4 (6)
	Health beliefs	2 (3)
	Intention or readiness	2 (3)
	Knowledge	15 (21)
	Participation in health care	2 (3)
	Pain	1 (1)
	Psychological	5 (7)
	Quality of life	2 (3)
	Satisfaction	1 (1)
	Vaccination	1 (1)
	Screening	10 (14)
**Study design**		
	Experimental	15 (21)
	Observational	32 (45)
	Qualitative	13 (18)
	Mixed methods	11 (16)

### Theme 2. Behavioral Intention to Use, Use, and Usefulness of Patient-Centered Technology

#### Electronic Health

Overall, 17 observational studies were identified for eHealth, with the majority targeting African Americans (n=9) (Multimedia Appendix 2) [68-84]. Most studies assessed cancer in general (n=8), with breast cancer being the most frequent single site focus (n=6). Studies found that the internet was the first source of cancer information, followed by health care providers, for Hispanics [68] and blacks [75]. In addition, email and Web-based information was preferred over mail [78]. Among a low-income population, Song et al [82] found that the internet was identified as the least relied upon source of general health information and cancer health information compared with *health professionals, family, and friends*. A majority of studies assessed technology use as an outcome (n=15).

In an early study, Gustafson et al [73] examined the feasibility of an interactive, computer-based system in reaching low-income, underserved women with breast cancer (N=229; n=85 African Americans) [85]. Low-income women were more likely to use and spend more time on the computer-based system compared with another population of more affluent women. In addition, low-income urban African Americans were more likely to use the system to access information and for health management services, whereas low-income whites were more likely to use communication services. The *Young Sisters Initiative: A Guide to a Better You!* program is a website designed for young breast cancer survivors. Using a mixed-methods approach, which included a postuse survey of 1442-site visitors (93% African American women), participants reportedly found value in using the website for reproductive and psychosocial information and support [86]. Chee et al [87] conducted a usability test and RCT pilot intervention to determine the efficacy of a culturally tailored registered nurse-moderated internet cancer support group. This study found positive effects on supportive care needs, psychological and physical symptoms, and quality of life.

In addition to personal computer use, the use of computer kiosks was explored among underserved populations. Kreuter et al [69] sought to understand the ideal placement (eg, beauty salons, churches, neighborhood health centers, laundromats, social service agencies, health fairs, and public libraries) to reach African American women for the purpose of providing tailored breast cancer information. This study found that only laundromats resulted in both frequent kiosk use and reaching high need populations (ie, a large proportion of users with no health insurance, unaware of where to get a mammogram, reporting no recent mammogram and barriers to getting one, and having little knowledge about breast cancer and mammography).

#### Mobile Health

We found 7 observational studies assessing mHealth (n=2) [88,89] or both mHealth and eHealth studies (n=5) [36-40]. Surveying 156 Hispanic and non-Hispanic rural women, Kratzke and Wilson [37,88] found that nearly 87% of study participants used cell phones, whereas 47% used text messaging as a means to communicate. Compared with non-Hispanic women, Hispanic women (n=36) were more receptive to breast cancer prevention voice messages and text messages. In another survey of Hispanic women (n=905), Dang et al [36] found that more than half of participants did not use the internet (58%) or email (64%), but a large proportion of participants used mobile phones (70%). In addition, 65% of all participants used text messages, with 45% wishing to receive mammogram reminders via SMS text message. Schoenberger et al used focus groups to assess the usage and acceptance of mobile communication technologies to provide cancer information among community health advisors (n=37) [90] and health ministry leaders (n=37) [91]. Among community health advisors, a majority of participants reported owning a mobile phone (89%) or a smartphone (67%) and 33% use text messaging as a means to communicate. All health ministry leaders reported owning a phone, whereas 85% reported using text messaging as a means to communicate.

#### Telemedicine

We identified 8 telemedicine studies using an observational design [92-99]. Of these, 3 studies focused on genetic counseling. Using surveys, McDonald et al [97] sought to understand the acceptability of telegenetics among Maine residents living in rurally remote areas. The most important characteristics of telegenetics models of care were perceived to be professional qualifications (92.2%) and one-on-one counseling (65.1%), whereas in-person and local counseling was ranked lower (51.8% and 52.1%, respectively).

Telemedicine was commonly used to provide psychosocial support. Rural lung, breast, and colorectal cancer patients reported a high level of satisfaction with a videophone-based intervention providing dignity psychotherapy [98]. Among a sample of Alaskan native breast cancer patients, an interactive audio and video telemedicine program providing medical consultation received overall high patient satisfaction [99]. In a sample of rural American Indian and Alaskan natives in Washington, cancer survivors were surveyed about their experiences with a telehealth cancer support group [93]. Members reported value in interacting with other cancer survivors and usefulness of the information presented. Specific topics of interest included nutrition during treatment as well as side effects of treatment.

### Theme 3. Perceptions and Satisfaction of Use of Patient-Centered Technologies

#### Mobile Health

We identified 5 qualitative studies related to mHealth (Multimedia Appendix 3) [91,100-103]. Qualitative studies primarily targeted black or African American (n=2) or Hispanic (n=2) populations and breast cancer (n=3) patients. These studies assessed outcomes related to content design and implementation (n=3) and usability or acceptability (n=2). Weaver et al [104] used focus groups to assess the perceptions of colorectal cancer screening text messages among a majority African American population (n=16; 62%). Although initially expressing reluctance to use personal technologies as a means to receive CRC information, participants responded favorably when shown sample text messages. Features that participants were interested in seeing with respect to text messages were personalized messages, content that was relevant to them, and messages that were positive and reassuring. Conversely, participants did not want to receive test results or bad news via text messages or content that included shorthand phrases or required complex replies. In a group of healthy African American men who received a prostate cancer screening educational intervention consisting of short text messages related to prostate cancer awareness, Le et al [105] found that 65% of the participants wished to continue receiving text messages pertaining to workshop reminders, postworkshop reinforcement, spiritual or motivational messages, and retention after completing the study.

Bravo et al [100] used semistructured interviews to assess the attitudes, acceptance, and usability of a breast cancer risk assessment tool accessed via tablet among underserved women seeking care at a safety net institution. A majority of women preferred the mobile app over a paper version of the assessment tool. All participants found the app easy to use.

#### Electronic Health

We identified 7 qualitative studies [106-112] and 7 mixed methods [86,87,113-117] related to eHealth. Qualitative studies primarily targeted black or African Americans (n=3) or diverse populations (n=2) and targeted breast (n=3) and prostate cancer (n=3) patients. These studies largely assessed outcomes related to usability or acceptability (n=3) and content design (n=2). For example, Berry et al [106] evaluated the usability of a Web-based decision aid designed to improve decision making among English-speaking Latino men (n=7) with localized prostate cancer. This eHealth intervention was tailored to participants’ personal factors (eg, personal characteristics, confidence in doctor, and influential people) and used expert recommendations to communicate health benefits and risks. Overall, participants rated the intervention with high acceptability. However, Berry et al found several usability issues related to *content comprehension* where Hispanic participants did not initially understand concepts until provided a short definition; *navigation issues* when answering multiple choice questions, using check boxes, typing responses with a keyboard, or clicking links to access external pages; and *sociocultural appropriateness* where some subgroups of the population (eg, Latino men in poverty) did not have computers at home and would therefore not use the app.

### Theme 4. Barriers and Facilitators to Use of Patient-Centered Technologies

#### Barriers

Text messaging was found to be beneficial as it provided a form of communication allowing for the quick dissemination of health information. The main barrier to text messaging was the lack of knowledge of how to text among some participants. [90,91] Increasing knowledge through education was found to be the most feasible solution. Another barrier to successful outcomes from text messaging [105] was read or receipt, wherein some participants did not remember receiving text messages. This problem could have arisen from technical issues and suggested the importance of incorporating a component into the intervention that verified that messages had been received.

#### Facilitators

Participants considered a complex mobile app to be easy and fun to use with easy to read text. In addition, participants were motivated to use the tool as it was new and innovative [100]. Although participants reported being unfamiliar with the technology (iPad) and experienced challenges with health literacy and security concerns, they had an interest in keeping up with technology [100]. Some suggestions identified when designing mHealth apps for this population include writing the content at the appropriate literacy levels, making instruction and assistance in using the mHealth app available, and minimizing the amount of new skillsets that participants will need to learn to use the mHealth app.

## Discussion

### Principal Findings

The purpose of this study was to review the current evidence on the use of cancer-specific patient-centered technologies among the underserved. Although the reviewed studies targeted various underserved populations including racial and ethnic minorities (eg, blacks or African Americans and Hispanics), low-income, and rural populations, we identified 2 cross-cutting issues that the literature suggests should be taken into account when implementing patient-centered technology interventions: (1) training in the use of patient-centered technologies and (2) tailoring patient-centered technologies to target populations.

The landscape of technology in our digital age is rapidly changing. This growth has led to several advances in health promotion from accessing health information digitally to using technology to track health and fitness [118]. In addition, the internet and mobile devices have become a prominent vehicle to reach diverse minority populations and deliver health information [23-28]. Use of the internet within the home is lower in individuals who are older, belong to a racial or ethnic minority group, are less educated, and have lower incomes [119,120]; however, the internet has become more accessible in many ways because of the proliferation of mobile devices. For example, blacks are more likely to access the internet with their mobile phone than their non-Hispanic white counterparts [119].

Much of the evidence related to mobile devices was devoted to the use of text messaging as a means to provide health information and facilitating behavior change. These results are promising given the consistent findings that underserved populations are receptive to the use of these technologies for cancer prevention and care purposes. Contributing factors to this growth, and the intention to use these technologies, may be traced back to the constructs of *social influence*, *price value*, and *habit*. According to the Pew Research Center, text messaging is being used by more than 90% of the population within each age group (100% of 18-29 years, 98% of 30-49 years, and 92% of >50 years) [121]. Due to this widespread use and already established habit of communicating with others, individuals are likely to adopt this form of technology to communicate with their social group. Due to the way in which this method of communication is ingrained into the day-to-day lives of individuals, the time cost of adopting these technologies is minimal because of their pervasiveness. In addition, using smartphones may be considered a low-cost alternative to accessing the internet compared with home internet.

Mobile devices provide a means to reach minority populations and offer the potential to reduce access issues with respect to health care and health information. However, barriers still exist that prevent the effective use of these technologies. In addition to creating opportunities to advance health promotion, the rapid growth in technology also presents several challenges. Of the most prominent challenges facing users, 1 is the pressure to remain updated with new technologies, their increasing *effort expectancy*, or the degree of ease associated with technology use. Clearly, realizing the full potential benefit of these technologies is dependent on their effective use. Although studies found that underserved populations are receptive to the use of patient-centered technologies, a recurrent challenge found in the literature was a lack of knowledge as to how to use new apps of the technology as well as the technology itself. These challenges should not be overlooked and range from receiving health information via text messages to using interactive iPads.

### Education and Training to Facilitate the Use of Patient-Centered Technologies

Some of these difficulties with use could be remedied by *facilitating conditions*, for example, providing a short training session at the same time the technology is introduced. Public libraries have been successful in improving decision making in accessing high-quality health information, reducing computer anxiety, and increasing computer interest and self-efficacy among older adults [122,123], and health care providers could leverage or learn from these community institutions. However, other technologies may require substantial modifications to the intervention to remove obstacles and barriers individuals may experience to facilitate their use. Interventions should incorporate usability and feasibility testing with target populations into their development process to identify unanticipated issues as well as appropriate training of target populations in the use of the technologies. Although such methods need to be applied efficiently to minimize their time and resource burden, up-front investment in such approaches can be the difference between a successful or failed implementation. In some cases, new technologies may not be a good fit with underserved populations, for example, low-income individuals may have insurmountable barriers to obtaining expensive new devices. Patients with disabilities, whether mental or physical, may not have the capacity to adapt to new technologies that require significant cognitive load or fine motor skills. In these cases, alternative communication channels may be necessary to deliver a desired behavioral or clinical intervention; we want to be careful to construct patient-centered technologies versus technology-centered patients.

### Tailoring to Facilitate the Use of Patient-Centered Technologies

When using interactive technologies (ie, computer-based media that enable users to access information and services of interest, control how the information is presented, and respond to information and messages in the mediated environment [124]), an important feature is the ability to tailor information to the recipients’ needs and interests [124,125]. It is necessary to consider the unique cultural norms and/or challenges of underserved populations when tailoring communication strategies. Robust methods to account for these differences in the design and implementation of technology interventions targeting specific groups is a key area in need of development. Hispanics may be better reached with technologies framed with health education content tailored to this population to improve both content comprehension and acceptance. For example, the use of telenovelas and soap operas is a novel approach that appeals to underserved Spanish-speaking women’s cultural norms and has been found to increase breast cancer screening knowledge and beliefs [57]. Similarly, a culturally tailored educational video, including a soap opera and physician recommendation segment made in Chinese was found to increase Chinese women’s intention to get screened for breast cancer, in addition to increasing their knowledge, perceived risk, and perceived benefits of screening [116].

Cultural competence is another strategy to reduce health and health care disparities that may be applied to the tailoring of patient-centered technologies. Cultural competence is defined as “...understanding the importance of social and cultural influences on patients’ health beliefs and behaviors; considering how these factors interact at multiple levels of the health care delivery system (eg, at the level of structural processes of care or clinical decision making); and, finally, devising interventions that take these issues into account to assure quality health care delivery to diverse patient populations” [126]. Tailoring patient-centered technologies to patients may help overcome sociocultural barriers to providing health care, one being a lack of culturally or linguistically appropriate health education materials [126]. By understanding unique differences among underserved groups, we can better understand how to reach each population, how they spend their time and use technology, and how different forms of technology may be used in different home and community settings. In addition, this approach allows researchers to tailor the technology based on who an individual is and how their identity is constructed before the technology is implemented.

### Directions for Future Research

#### Digital Divide

The use of patient-centered technologies may be seen as a means to reach underserved populations; however, there are several concerns within the health care research community related to their use. Of particular interest is the decreased access of technologies among racial and ethnic minorities, persons with disabilities, rural populations, older populations (including veterans), and individuals with lower socioeconomic status; a phenomenon commonly referred to as the digital divide [127,128]. More research needs to assess the health information needs of these underserved populations and how they prefer to receive health information. When the use of technology may not be appropriate for providing patient-centered care, the use of other tailored interventions may be more successful.

#### Underrepresented Cancers and Underserved Populations

According to the American Cancer Society, the most prevalent cancer among men is prostate cancer, whereas breast cancer is the most common cancer among women [129]. In addition, lung and colorectal cancer comprise the second and third most common cancer in both men and women [129]. Although several studies have targeted breast cancer, colorectal, and prostate cancer, only a few studies have targeted other cancers. Future research on the use of patient-centered technologies among underserved populations should focus on prevalent cancers, which are underrepresented in the HIT literature such as lung cancer. Importantly, lung cancer is the leading cause of cancer deaths in black men and women, and black men have higher rates of lung cancer than their non-Hispanic white counterparts [130]. Furthermore, less prevalent cancers have not seen the same level of technology development targeting their unique clinical needs. Although some cancers are less prevalent in the general population, they may disproportionately impact underserved populations. For example, Hispanic men experience liver cancer incidence rates twice that of non-Hispanic white men [131]. Liver cancer also serves as the second leading cause of cancer deaths. Cancers of the female reproductive system are also underrepresented, including ovarian and cervical cancers. African American women are diagnosed with more advanced stages of ovarian cancer and have lower survival rates compared with their white counterparts. In addition, there are underserved populations that have received little attention in the current literature. Studies predominantly targeted black or African Americans and Hispanic populations, and some underrepresented populations include American Indians or Alaskan Natives and Asian populations.

#### Underused Technologies

The current evidence with respect to barriers and facilitators to the use of patient-centered technologies may be used to guide the development of other technologies, such as PHRs, which did not appear in our review. PHRs have been defined as “an Internet-based set of tools that allows people to access and coordinate their lifelong health information and make appropriate parts of it available to those who need it.” PHRs can be tethered (connected) to eHealth records and provide patients with an asynchronous platform to access and update their medical health record data and engage with their health care team [9,132,133]. As an example, the unique challenges we identified among Hispanics, including issues related to tailored educational content and comprehension, may be used to modify patient portals within practices serving a Hispanic community.

#### Patient-Provider Communication and Shared Decision Making

Our review also highlights the paucity of research regarding how health information technology can improve communication and shared decision making (SDM) between individuals from vulnerable populations and their health care providers. Although increasing provider communication is important in building trust and improving chronic disease management [4], SDM bridges gaps in knowledge, tailors medical and health decisions to patient preferences, as well as increases patient adherence to treatment and improves health outcomes [134]. More research should focus on the use of these technologies to support providers in delivering information to patients on cancer treatment options as well as describe the advantages and disadvantages of different approaches to technology design and implementation.

#### Precision Medicine

By tailoring content to targeted populations, patient-centered technologies have the potential to facilitate the provision of precision medicine among underserved populations. Precision medicine focuses on the “...prevention and treatment strategies that take individual variability into account” [135]. Interactive technologies such as social media may take advantage of predictive algorithms to tailor the care of individuals to patients based not only on their genetic but also on their social identities.

### Limitations

Our study has several limitations. Of the potential limitations, 1 is that our search strategy may not have captured all potential papers meeting our inclusion criteria. To minimize this limitation, we implemented a snowball search method in which we reviewed the references of all included studies for additional citations. Another limitation of our study is that because of the heterogeneity in study design and types of outcomes evaluated, we were unable to aggregate findings in the manner of a meta-analysis. Finally, the included papers may be subject to publication bias as studies that report negative findings are less likely to be published.

### Conclusions

There is a growing body of literature exploring patient-centered technology and its influence on the care of underserved populations. Despite the potential of patient-centered technologies and their acceptance among underserved populations, challenges still exist with respect to their effective use and usability. With technology changing at an exceedingly rapid pace, more training needs to be provided to ensure these underserved groups are able to effectively use new and emerging technologies. In addition, tailoring these technologies to unique cultural norms will be critical to facilitating their effective use.

## Appendix

Multimedia Appendix 1 Description and findings of experimental studies included in this review.

Multimedia Appendix 2 Description and findings of observational studies included in this review.

Multimedia Appendix 3 Description and findings of qualitative and mixed-methods studies included in this review.

## Abbreviations

eHealth: electronic health
HIT: health information technology
mHealth: mobile health
PHR: personal health record
RCT: randomized controlled trial
SDM: shared decision making
SMS: short message service
UTAUT: unified theory of acceptance and use of technology
