# Supplementary material for: The Use of Cancer-Specific Patient-Centered Technologies Among Underserved Populations in the United States: Systematic Review
Source: J Med Internet Res. 2019 Apr 23;21(4):e10256. doi: 10.2196/10256 (PMC6658273; doi:10.2196/10256)
Supplement: Multimedia Appendix 1 [file jmir_v21i4e10256_app1.pdf]

Multimedia Appendix 1. Description and findings of experimental studies included in this review (n=15).

Electronic Health Studies (n=10)

Author (year)	Description of Technology	Underserved population (Sample Size)	Cancer	Outcomes Assessed	Major Findings
Champion (2006)	Interactive computer-assisted program (tailored)	Black or African American (n=344)	Breast	Screening	Intervention group had greater adherence to mammography (40.0%) compared with video (24.6%) and pamphlet (32.1%).
Christy (2013)	Computer-tailored colorectal cancer screening intervention (tailored)	Black or African American (n=693)	Colorectal	Communication	Intervention group reported higher level of discussion with provider than participants using brochure (63% vs 48%, odds ratio [OR] 1.81, *P*<.001).
Ellison (2008)	Web-based decision aid (tailored)	Black or African American (n=87)	Prostate	Knowledge	Knowledge scores (benefits, limitations, and risks of screening) were higher in intervention group compared with usual care.
Greiner (2014)	Touchscreen computer-delivered implementation intentions intervention (tailored)	Diverse, low-income population (n=470)	Colorectal	Screening	Intervention group had higher odds of completing CRC screening (OR 1.83).
Gustafson (2005)	eHealth system providing information, decision making, and support (tailored)	Low-income (n=246)	Breast	Knowledge<br>Participation in Health Care<br>Psychological	Intervention group participants were found to be more competent at seeking information, more comfortable participating in health care, and had significantly better social support and information competence. The eHealth intervention provided more benefit to underserved populations.
Jibaja (2000)	Tailored, interactive soap opera (tailored)	Hispanic (n=178)	Breast	Health Beliefs<br>Knowledge	Intervention group participants showed significant increases in breast cancer screening knowledge and beliefs.

Electronic Health Studies (n=10) continued

Author (year)	Description of Technology	Underserved population (Sample Size)	Cancer	Outcomes Assessed	Major Findings
Miller (2011)	Web-based multimedia colorectal cancer screening decision aid	Black or African American (n=264)	Colorectal	Decision Making<br>Intention or Readiness	Decision aid participants more likely to have CRC screening preference (84% vs 55%, *P*<.001) and increased readiness to receive screening (52% vs 20%, *P*<.001).
Rawl (2012)	Computer-delivered tailored intervention to increase CRC screening (tailored)	Black or African American (n=556)	Colorectal	Health Beliefs<br>Knowledge	Intervention increased CRC knowledge scores (*P*=.005), perceived CRC risk scores, fecal occult blood test barriers scores, and colonoscopy benefit.
Russell (2010)	Interactive computer program and lay health advisor intervention (tailored)	Black or African American (n=181)	Breast	Screening	Intervention group had increased screening compared with brochure group.
Schroy (2011)	Computer-based decision aid for CRC screening	Predominantly Black or African American (n=665)	Colorectal	Decision Making<br>Knowledge	Shared decision making, knowledge scores, and intention scores were significantly higher for intervention groups consisting of the interactive computer-based decision aid compared with control groups.

Mobile Health Studies (n=3)

Author (year)	Description of Technology	Underserved population (Sample Size)	Cancer	Outcomes Assessed	Major Findings
Fernandez (2015)	Tailored interactive multimedia intervention (TIMI) delivered on tablet computers (tailored)	Hispanic (n=665)	Colorectal	Knowledge<br>Psychological<br>Screening	No significant difference was found.
Lee (2016)	Culturally tailored mobile health intervention to increase Human Papillomavirus (HPV) vaccination (tailored)	Asian (n=30)	Cervical (HPV)	Knowledge<br>Vaccination	Significant increases in HPV knowledge. Uptake of HPV vaccine was initiated by 30% of participants.
Yanez (2015)	Technology-assisted psychosocial intervention	Black African American (n=74)	Prostate	Psychological	The technology-based intervention was found to be feasible (>85% retention and >70% attendance rates) and acceptable among participants. Participants reported significant reductions in depressive symptoms and improvements in relaxation self-efficacy.

Experimental Telehealth Studies (n=2)

Author (year)	Description of Technology	Underserved population (Sample Size)	Cancer	Outcomes Assessed	Major Findings
Buchanon (2015)	Telegenetics cancer genetic counseling	Rural (n=162)	Cancer (not specific)	Satisfaction	Patient satisfaction did not differ between telegenetics and in-person counseling group.
Kroenke (2010)	Telecare management with automated home-based symptom monitoring by interactive voice recording or internet	Rural (n=405)	Cancer (not specific)	Pain<br>Psychological	Intervention participants showed significantly greater improvements in pain and depression.
