# Supplementary material for: The Use of Cancer-Specific Patient-Centered Technologies Among Underserved Populations in the United States: Systematic Review
Source: J Med Internet Res. 2019 Apr 23;21(4):e10256. doi: 10.2196/10256 (PMC6658273; doi:10.2196/10256)
Supplement: Multimedia Appendix 2 [file jmir_v21i4e10256_app2.pdf]

Multimedia Appendix 2. Description and findings of observational studies included in this review (n=32).

Electronic Health Studies (n=17)

Author (year)	Description of Technology	Underserved population (Sample Size)	Cancer	Outcomes Assessed	UTAUT-2 Constructs	Major Findings
Allen (2009)	Computer-tailored intervention for prostate cancer screening (tailored)	Black or African American (n=108)	Prostate	Decision making<br>Knowledge	Performance expectancy	Significant improvements were found in knowledge, decision self-efficacy, and decisional conflict.
Eddens (2009)	Web-based stories of cancer survivors	Diverse population (n=106)	Cancer (not specific)	Use	Performance expectancy<br>Social influence	Minorities accounted for 9.8% of Web-based stories of cancer survivors.
Giordano (2015)	Cancer information seeking practices	Hispanic (n=622)	Cancer (not specific)	Use	Performance expectancy	The leading sources of cancer information came from the internet (47%, n=105), followed by health care providers (26%, n=60).
Greaney (2014)	Web use for behavioral risk factors	Black or African American (n=2440; 27% Black or AA)	Cancer (not specific)	Use	Facilitating conditions	Whites were more likely to pick Web-based material than black participants.
Gustafson (2005)	Comprehensive health enhancement support system (CHESS)	Low-income women (Rural [n=144] or Black or African American (n=85)	Breast	Use	Performance expectancy<br>Facilitating conditions	Low-income subjects logged on and spent more time on CHESS Urban African Americans used information and analysis services more and communication services less than rural whites.
Helft (2005)	Use of the internet to obtain cancer information	Black or African American (n=200; 45% Black or AA)	Cancer (not specific)	Use	Performance expectancy<br>Facilitating conditions	10% of participants used the internet to obtain cancer information.<br>Lack of internet access was most common barrier Less education, African American race, and female sex were associated with lower estimates of the accuracy of internet information.
Kelly (2009)	Information seeking preferences	Black or African American (n=101; 40.6% Black or AA)	Cancer (not specific)	Use	Performance expectancy<br>Effort expectancy	The internet was the most common (43%) first source sought for hereditary cancer information, followed by physicians (23%).

[a]UTAUT: unified theory of acceptance and use of technology.

Electronic Health Studies (n=17) continued

Author (year)	Description of Technology	Underserved population (Sample Size)	Cancer	Outcomes Assessed	UTAUT-2 Constructs	Major Findings
Kreuter (2006)	Computer kiosks for breast cancer education (tailored)	Rural black African American (n=4527)	Breast	Use<br>Knowledge	Performance expectancy<br>Effort expectancy<br>Facilitating conditions	Among different community settings, kiosk use was higher for those located in laundromats (14.0 users per day), followed by neighborhood health centers (9.9) and churches (9.3) Kiosk use was associated with significant improvements in knowledge, decision self-efficacy, and decisional conflict.
Kreuter (2008)	Computer kiosks for breast cancer education (tailored)	Rural black or African American (n=10,306)	Breast	Use<br>Knowledge	Performance expectancy<br>Effort expectancy<br>Facilitating conditions	The placing of kiosks in laundromats (compared with other settings, including beauty salons, churches, neighborhood health centers, health fairs, and libraries) was found to provide high reach and access to users with no insurance who were unaware of where to get a mammogram, who reported no recent mammogram, and had little knowledge of breast cancer and screening.
Monnier (2002)	Internet-based cancer services website	Asian or rural (n=319)	Cancer (not specific)	Knowledge	Performance expectancy<br>Habit	Minorities, older individuals, and less educated individuals were less likely to have knowledge of and to have used the internet to access cancer information.
Paxton (2014)	Preferences for physical activity interventions among African American breast cancer survivors	Asian (n=475)	Breast	Participation in Health Care	Performance expectancy<br>Effort expectancy	Participants expressed the greatest interest in email (50%) or Web-based (48%) programs over mail (45%), group (39%), and telephone (10%).

[a]UTAUT: unified theory of acceptance and use of technology.

Electronic Health Studies (n=17) continued

Author (year)	Description of Technology	Underserved population (Sample Size)	Cancer	Outcomes Assessed	UTAUT-2 Constructs	Major Findings
Quin (2010)	Internet lung cancer support	Rural (n=597)	Lung	Use	Performance expectancy<br>Effort expectancy<br>Facilitating conditions	Overall, 343 patients (57.4%) had a home computer and 299 (50.1%) had home internet service Average internet use was 8.5 h per week, and 225 patients used the internet for health information.<br>In total, 92 patients had lung cancer and 10 indicated interest in onsite support services, whereas 37 expressed interest in internet-based support.
Ramirez (2002)	Computer-based cancer education	Hispanic (n=292)	Cancer (not specific)	Usefulness<br>Decision-Making	Performance expectancy	CancerHelp did not help Latino patients and family members make decisions about their care more than other ethnic groups.
Santos (2014)	Web-based training system for community health advisors	Black or African American (n=28)	Cancer (not specific)	Usefulness<br>Usability or acceptability	Performance expectancy<br>Effort expectancy	The Web-based portal allowed CHAs to log in and view training videos that lasted an average of 26 days.<br>During this period, community health advisors required little technical assistance suggesting a Web-based system as a feasible method for training CHAs.
Song (2015)	Technology use for at-risk prostate cancer	Low-income (n=90)	Prostate	Use	Performance expectancy	The internet was the least relied on source of information Participants primarily relied on health professionals, family, and friends.

[a]UTAUT: unified theory of acceptance and use of technology.

Electronic Health Studies (n=17) continued

Author (year)	Description of Technology	Underserved population (Sample Size)	Cancer	Outcomes Assessed	UTAUT-2 Constructs	Major Findings
Wise (2008)	Web-based narrative and didactic information	Black or African American (n=353 [31.4% Black or AA])	Breast	Use<br>Participation in Health Care	Performance expectancy	Web-based narrative and didactic information had positive effects on health care participation Effects of both were significantly greater for African Americans.
Yi (2010)	Internet use among Chinese cancer survivors	Asian (n=72)	Breast	Use<br>Communication	Performance expectancy	56% used the internet for health information; only 9% used email or visited a website to communicate with a doctor's office or get Web-based provider advice.

[a]UTAUT: unified theory of acceptance and use of technology.

Mobile Health Studies (n=2)

Author (year)	Description of Technology	Underserved population (Sample Size)	Cancer	Outcomes Assessed	UTAUT-2 Constructs	Major Findings
Kratzke (2014)	Cancer prevention information seeking	Rural (n=156)	Breast	Use<br>Knowledge	Performance expectancy<br>Effort expectancy<br>Facilitating conditions	Television, magazines, and internet were the most frequent information sources.<br>Providers were the most frequent interpersonal information source Nearly 87% used cell phones and 47% used short message service (SMS) text messaging.<br>Hispanic women were more likely to desire breast cancer prevention cell voice messages (*P*<.001) and SMS text messages (*P*=.001) compared with non-Hispanic women.
Purnell (2014)	Mobile technology for cancer prevention	Low-income black or African American (n=1898)	Cancer (not specific)	Use	Performance expectancy<br>Effort expectancy<br>Facilitating conditions<br>Habit	Three-fourths (74%) of study participants owned a cell phone and 19% owned a smartphone SMS text messaging was the most popular use.

[a]UTAUT: unified theory of acceptance and use of technology.

Electronic Health and Mobile Health Studies (n=5)

Author (year)	Description of Technology	Underserved population (Sample Size)	Cancer	Outcomes Assessed	UTAUT-2 Constructs	Major Findings
Dang (2013)	Internet, email, and short message service (SMS) text messaging for promotion of breast health	Hispanic women (n=905)	Breast	Use<br>Knowledge	Performance expectancy<br>Habit	Survey found that most Hispanic women in sample never use the internet (58%) or email (64%), whereas 70% have mobile phones and 65% use SMS text messaging daily.<br>Overall, 45% of participants wish they received a mammogram reminder SMS text message.
Kratzke (2013)	Breast cancer prevention information seeking through apps and SMS text messages	Rural women (n=157)	Breast	Use<br>Usability or readiness	Performance expectancy	In total, 87% of participants used cell phones, whereas 20% had an interest in receiving breast cancer prevention SMS text messages.<br>Overall, 47% of participants used SMS text messaging, whereas 36% had an interest in receiving breast cancer prevention SMS text messages and 37% had an interest in receiving mammogram reminders.
Kratzke (2014)	Breast cancer prevention information seeking through apps and SMS text messages	Hispanic college women (n=546)	Breast	Use<br>Usability or readiness	Performance expectancy	Overall, 44% of participants used the internet for active breast cancer prevention information seeking Participants also desired breast cancer prevention apps (54%) and SMS text messages (51%).<br>Hispanic women were more likely to desire breast cancer prevention apps compared with non-Hispanic women.

[a]UTAUT: unified theory of acceptance and use of technology.

Electronic Health and Mobile Health Studies (n=5) continued

Author (year)	Description of Technology	Underserved population (Sample Size)	Cancer	Outcomes Assessed	UTAUT-2 Constructs	Major Findings
Haughton (2005)	Access to communication technologies	Black or African American women (n=1227)	Breast	Use<br>Knowledge	Performance expectancy<br>Facilitating conditions	Among a low-income African American population of women, <10% reported email access, whereas 26% reported cell phone or pager access. Cell phone and pager access was positively associated with mammography knowledge.
Robinson (2015)	Communication technology access and preferences among Alaskan natives	Alaskan natives (n=673)	Colorectal	Use	Performance expectancy<br>Effort expectancy<br>Facilitating conditions	A majority of respondents reported computer access (98%), email access (97%), and mobile phone use (94%).<br>Overall, 60% of mobile phone users had internet access through their phones. Alaskan native women were significantly more likely to have computer access (*P*=.01) and use email (*P*=.005).<br>No significant gender differences were found in mobile phone access to the internet or short message service text messaging. Older respondents (aged 65-80 years) were significantly less likely to anticipate using the internet to schedule appointments, refill medications, or communicate with their health care providers.

[a]UTAUT: unified theory of acceptance and use of technology.

Telehealth Studies (n=8)

Author (year)	Description of Technology	Underserved population (Sample Size)	Cancer	Outcomes Assessed	UTAUT-2 Constructs	Major Findings
Allen (1995)	Interactive videoconferencing (tailored)	Rural (n=39)	Cancer (not specific)	Satisfaction	Performance expectancy	Participants reported relatively high levels of satisfaction with telemedicine at initial clinic visits and follow-up visits.<br><br>Rural cancer patients may be satisfied with using telemedicine to see oncologists, at least on an occasional basis.
Doorenbos (2010)	Telehealth support group services	Rural American Indian or Alaska Native (n=32)	Cancer (not specific)	Satisfaction<br><br>Usability or acceptability	Performance expectancy<br><br>Social influence	Cancer patients valued the opportunity to interact with other American Indian and Alaska Native cancer survivors living in remote locations as they felt they were no longer alone in their cancer experiences.<br><br>Patients also found the information useful and the usefulness of the information presented.
Hitt (2013)	Telecolposcopy	Rural women (n=1504)	Cervical	Screening	Performance expectancy<br><br>Price value	This project provided complex specialty gynecological services using telemedicine technology to 1504 patients (1812 visits). Telemedical cervical cancer screening provided a cost-effective way to overcome geographic barriers to care while producing results comparable with traditional examinations.
Hitt (2016)	Telecolposcopy	Rural women (n=940)	Cervical	Screening	Performance expectancy<br><br>Price value	The telecolposcopy program was found to be sustainable statewide. Patients were willing to participate as it increased access to care and reduced travel time and costs associated with care for rural, underserved women.

[a]UTAUT: unified theory of acceptance and use of technology.

Telehealth Studies (n=8) continued

Author (year)	Description of Technology	Underserved population (Sample Size)	Cancer	Outcomes Assessed	UTAUT-2 Constructs	Major Findings
McDonald (2014)	Telemedicine cancer genetic counseling	Rural (n=149)	Cancer (not specific)	Usability or acceptability	Performance expectancy	This study found that the most important characteristics of telegenetics models of care were perceived to be professional qualifications (92.2%) and one-on-one counseling (65.1%), whereas in-person and local counseling was ranked lower (51.8% and 52.1%, respectively).
Mette (2016)	Cancer genetic counseling video teleconferencing (tailored)	Hispanic (n=119)	Cancer (not specific)	Satisfaction<br>Usability or acceptability	Performance expectancy	This underserved Hispanic population reported very high satisfaction and acceptability of video teleconferencing to provide cancer risk assessment.
Passik (2004)	Dignity psychotherapy delivered telemedicine	Rural (n=8)	Cancer (not specific)	Satisfaction<br>Usefulness	Performance expectancy	Participants reported overall benefit and high levels of satisfaction from the intervention. This work shows the use of telemedicine to deliver Dignity psychotherapy to individuals too ill to leave their homes or are located in rural locations may be a feasible way to help patients dying at home.
Pruthi (2013)	Telemedicine-based counseling program	Alaskan native women (n=15)	Breast	Satisfaction	Performance expectancy	This study found a 98% overall patient satisfaction with the interactive audio and visual telemedicine program.

[a]UTAUT: unified theory of acceptance and use of technology.
