# Supplementary material for: The Use of Cancer-Specific Patient-Centered Technologies Among Underserved Populations in the United States: Systematic Review
Source: J Med Internet Res. 2019 Apr 23;21(4):e10256. doi: 10.2196/10256 (PMC6658273; doi:10.2196/10256)
Supplement: Multimedia Appendix 3 [file jmir_v21i4e10256_app3.pdf]

Multimedia Appendix 3. Description and findings of qualitative and mixed-methods studies included in this review (n=24).

Electronic Health Qualitative Studies (n=7)

Author (year)	Description of Technology	Underserved population (Sample Size)	Cancer	Outcomes Assessed	UTAUT-2 Constructs	Major Findings
Berry (2015)	Usability evaluation of a prostate decision aid for Latino men (tailored)	Hispanic (n=7)	Prostate	Usefulness<br>Usability or acceptability	Performance expectancy<br>Effort expectancy	Study identified barriers to effective decision support in intervention.<br>The population experienced issues with content comprehension, navigation issues, and a lack of sociocultural appropriateness Solutions to address these issues include framing and tailoring the technology to Latino men and guide them through the technology use.
Im (2016)	Culturally tailored internet cancer support (tailored)	Asian (n=56)	Breast	Usability or acceptability	Effort expectancy<br>Facilitating conditions	Identified several development and implementation issues.<br>These included difficulties in recruitment and retention (limited Asian Web-based communities) and difficulties in using multiple languages (translation issues).
Jaja (2010)	Prostate cancer Web-based decision support system (tailored)	Black or African American (n=12)	Prostate	Usability or acceptability	Performance expectancy	The most common usability issues experienced by participants included lack of completeness of information content and the comprehensibility of short message service text message and graphics.
Joseph (2010)	Computer-based cancer risk education intervention tool (CREdIT)	Diverse population (n=11)	Ovarian	Usability or acceptability<br>Design or implementation	Performance expectancy<br>Effort expectancy	Genetic counseling and testing for breast cancer was a new to most participants CREdIT provided new information and corrected misunderstandings about inheritance of disease. Although CREdIT increased knowledge, misunderstandings of cancer risk still persisted among some participants.

[a]UTAUT: unified theory of acceptance and use of technology.

Electronic Health Qualitative Studies (n=7) continued

Author (year)	Description of Technology	Underserved population (Sample Size)	Cancer	Outcomes Assessed	UTAUT-2 Constructs	Major Findings
May (2016)	Preferred health information sources for colorectal cancer screening in African Americans	Black or African American (n=38)	Colorectal	Design or implementation	Performance expectancy<br>Effort expectancy<br>Social influence	Common sources of health information included media and medical providers Suggested dissemination of CRC screening education included the internet and interventions should include culturally specific information.
McTavish (2003)	Computer-mediated breast cancer discussion group (tailored)	Diverse population (n=121)	Breast	Use	Performance expectancy	Blacks and whites differed in their use of the Comprehensive health enhancement support system (CHESS) system Although blacks used it less frequently, their use was more instrumental and focused on the management of breast cancer.
Owens (2015)	Computer-based prostate cancer decision aid	Black or African American (n=39)	Prostate	Design or implementation	Performance expectancy<br>Effort expectancy<br>Habit	Participants were generally knowledgeable about prostate cancer, but few engaged in informed decision making with their doctor or were informed about the associated risks of screening.<br>Most participants used technology on a daily basis for health information seeking and were open to a novel computer-based intervention if the system was easy to use and its animated avatars were culturally appropriate.

[a]UTAUT: unified theory of acceptance and use of technology.

Mobile Health Qualitative Studies (n=6)

Author (year)	Description of Technology	Underserved population (Sample Size)	Cancer	Outcomes Assessed	UTAUT-2 Constructs	Major Findings
Arvey (2012)	Tablet-based interactive computer program (tailored)	Hispanic (n=5)	Colorectal	Design or implementation	Effort expectancy<br>Habit	Technical issues arose as a result of inexperience with technologies that led to feelings of anxiety, discomfort, and lack of control.
Banas (2016)	Peer-to-peer mHealth app to connect Spanish patients	Hispanic (n=31)	Breast	Design or implementation	Performance expectancy<br>Facilitating conditions<br>Social influence	Participants would be receptive to an intervention that offered not only information and support on cancer and treatment effects but also incorporate Spanish content and connect with others who had gone through a similar experience.
Bravo (2014)	Breast Health Questionnaire app (breast cancer risk assessment)	Low-income population (n=15)	Breast	Usability or acceptability	Performance expectancy<br>Effort expectancy<br>Facilitating conditions<br>Hedonic motivation	Barriers experienced by participants included unfamiliarity with iPad, low health literacy, and security concerns Facilitators to the use of the breast cancer risk assessment tool included tailoring content by literacy level and provide assistance and instruction in iPad use.
Schoenberger (2015)	Disseminating cancer information to Health Ministry leaders through short message service (SMS) text message	Black or African American (n=37)	Cancer (not specific)	Use<br>Usability or acceptability	Effort expectancy<br>Habit	Participants experienced several challenges, including a lack of knowledge of how to SMS text message and experienced issues receiving multiple messages.<br>Solutions identified included increasing knowledge through education.

[a]UTAUT: unified theory of acceptance and use of technology.

Mobile Health Qualitative Studies (n=6) continued

Author (year)	Description of Technology	Underserved population (Sample Size)	Cancer	Outcomes Assessed	UTAUT-2 Constructs	Major Findings
Smith (2016)	Mobile cancer prevention app	Black or African American (n=12)	Breast	Design or implementation	Performance expectancy<br>Effort expectancy	Describes a community-engaged process to develop mobile Web-based app that is theory driven Information elicited from African American breast cancer survivors fell into 3 major categories: (1) perceptions about modifiable risk factors, (2) strategies related to adherence to cancer prevention guidelines, and (3) app components to address barriers to adherence.
Weaver (2015)	SMS text messages to increase colorectal cancer screening test completion	Predominantly black or African American (n=26)	Colorectal	Use<br>Design or implementation	Performance expectancy	Participants initially expressed reluctance to use personal technologies as a means to receive CRC information; participants responded favorably when shown sample SMS text messages Features that participants were interested in seeing with respect to SMS text messages were personalized messages, content that was relevant to them, and messages that were positive and reassuring.

[a]UTAUT: unified theory of acceptance and use of technology.

Electronic Health Mixed Methods Studies (n=7)

Author (year)	Description of Technology	Underserved population (Sample Size)	Cancer	Outcomes Assessed	UTAUT-2 Constructs	Major Findings
Chee (2016)	Tailored internet cancer support group (tailored)	Asian (n=65)	Breast	Performance expectancy<br>Effort expectancy	Usability or acceptability<br>Physical<br>Psychological<br>Quality of life	Usability testing found that users positively evaluated the internet cancer support group on display, educational contents, and user-friendliness.<br>The support group also significantly improved physical symptoms, psychological symptoms, and quality of life.
Johnson-Turbes (2015)	Web-based psychosocial and reproductive health support intervention (tailored)	Black or African American (n=1442)	Breast	Performance expectancy	Usefulness	A majority of participants reported being very or somewhat satisfied (75%) with the program, whereas 70% found the program somewhat or very useful.
Kukafka (2015)	Web-based decision aid and risk assessment tool (tailored)	Multiethnic women (n=34; 23.5% Black or AA; 61.8% Hispanic)	Breast	Performance expectancy	Decision-making	Use of the Web-based decision aid and risk assessment tool improved the accuracy of perceived breast cancer risk among participants.
Lee (2014)	Culturally tailored DVD intervention (tailored)	Asian (Vietnamese)	Colorectal	Usability or acceptability<br>Knowledge<br>Screening	Performance expectancy<br>Facilitating conditions	No significant difference in screening was found between intervention and control group. Both groups showed increased knowledge about CRC 1 month post intervention Qualitative focus group findings revealed that the use of the DVD was an effective method of communicating information among this population and would help promote screening.

[a]UTAUT: unified theory of acceptance and use of technology.

Electronic Health Mixed Methods Studies (n=7) continued

Author (year)	Description of Technology	Underserved population (Sample Size)	Cancer	Outcomes Assessed	UTAUT-2 Constructs	Major Findings
Menon (2008)	Computer-based, touch screen educational program to increase colorectal cancer screening (tailored)	Predominantly black or African American (n=199)	Colorectal	Usefulness<br>Usability or acceptability<br>Screening	Performance expectancy	A majority of participants found the intervention useful (91%).<br>The intervention also aided participants in the decision to get screened (80%) and helped them overcome barriers (49%).<br>Overall, 68% of participants thought it raised new concerns about cancer, whereas only 30% said it made them worry about cancer.
Owens (2016)	Prostate cancer decision aid for African American men	Black or African American (n=39)	Prostate	Use<br>Usefulness<br>Usability or acceptability	Effort expectancy<br>Habit	Most patients in sample used interactive communication technologies on a daily basis for purposes that included information seeking Most participants were open to novel computer-based interventions if they were easy to use and were culturally appropriate.
Wang (2008)	Culturally tailored video including soap opera and physician-recommendation segment (tailored)	Asian (Chinese) (n=72)	Breast	Use<br>Intention or readiness<br>Knowledge	Performance expectancy<br>Social influence	The culturally tailored video significantly increased Chinese women's screening intentions, knowledge, perceived risk for breast cancer, and perceived benefits of mammography.

[a]UTAUT: unified theory of acceptance and use of technology.

Mobile Health Mixed Methods Studies (n=4)

Author (year)	Description of Technology	Underserved population (Sample Size)	Cancer	Outcomes Assessed	UTAUT-2 Constructs	Major Findings
Lee (2014)	Mobile phone short message service (SMS) text message–based cervical cancer screening intervention (tailored)	Asian (Korean American) women (n=30)	Cervical	Usability or acceptability<br>Knowledge<br>Screening	Performance expectancy	SMS text messages significantly increased participants' knowledge of cervical cancer and screening guidelines.<br>A majority of participants reported satisfaction with the intervention (83%) and 97% of participants would recommend the intervention to others (acceptability or feasibility).
Le (2015)	SMS text messaging use in an African American prostate cancer educational intervention	Black or African American (n=23 for focus groups; n=288 for intervention)	Prostate	Use<br>Usability or acceptability	Performance expectancy<br>Effort expectancy	Overall, 65% of the participants wished to continue receiving SMS text messages pertaining to workshop reminders, postworkshop reinforcement, spiritual or motivational messages, and retention after completing the study. However, more than one-third of the study participants did not recall receiving the text messages.
Oakley-Girvan (2016)	SMS text messaging intervention used to improve abnormal follow-up of mammograms	Hispanic (n=29)	Breast	Screening	Performance expectancy	SMS text messaging decreased time for receipt of an abnormal mammogram for Latina women (median of 23 days to return for follow-up for intervention group compared with 59 days for delayed intervention group).

[a]UTAUT: unified theory of acceptance and use of technology.

Mobile Health Mixed Methods Studies (n=4) continued

Author (year)	Description of Technology	Underserved population (Sample Size)	Cancer	Outcomes Assessed	UTAUT-2 Constructs	Major Findings
Schoenberger (2013)	Community health advisor use of short message service (SMS) text messages to access health information	Black or African American (n=37 for focus groups; n=77 for surveys)	Cancer (not specific)	Use<br>Communication	Performance expectancy<br>Effort expectancy<br>Facilitating conditions<br>Habit<br>Price value	Overall, 89% of focus group participants self-report owning a mobile phone with 24% owning a smart phone and 67% having a SMS text-messaging plan. SMS text messaging benefits included it being an alternative form of communication, a quick method for disseminating information, and privacy of communication. Conversely, barriers included cost and not knowing how to SMS text message.

[a]UTAUT: unified theory of acceptance and use of technology.
